# The Prevalence of Comorbidities in Individuals with Periodontitis in a Private Periodontal Referral Practice

**DOI:** 10.3390/jcm13237410

**Published:** 2024-12-05

**Authors:** Nicky G. F. M. Beukers, Bruno G. Loos, Geert J. M. G. van der Heijden, Elena Stamatelou, Athanasios Angelakis, Naichuan Su

**Affiliations:** 1 Department of Periodontology, Academic Centre for Dentistry Amsterdam, University of Amsterdam and Vrije Universiteit Amsterdam, Gustav Mahlerlaan 3004, 1081 LA Amsterdam, The Netherlands; 2 Praktijk voor Parodontologie en Implantologie Nijmegen, Sint Annastraat 255, 6525 GR Nijmegen, The Netherlands; 3 Praktijk voor Parodontologie en Implantologie Horst, Stationsstraat 50, 5961 HS Horst, The Netherlands; 4 Department of Oral Public Health, Academic Centre for Dentistry Amsterdam, University of Amsterdam and Vrije Universiteit Amsterdam, Gustav Mahlerlaan 3004, 1081 LA Amsterdam, The Netherlands; 5 Department of Epidemiology and Data Science, Amsterdam University Medical Centers, Meibergdreef 9, 1105 AZ Amsterdam, The Netherlands; 6 Methodology and Digital Health, Amsterdam Public Health Research Institute, Meibergdreef 9, 1105 AZ Amsterdam, The Netherlands; 7 Data Science Center, University of Amsterdam, Singel 425, 1012 WP Amsterdam, The Netherlands

**Keywords:** cluster analysis, comorbidity, epidemiology, hypergraph network analysis, multimorbidity, periodontal disease

## Abstract

**Objectives:** Periodontitis (PD) patients frequently suffer from comorbidities, necessitating increased attention to disease management and monitoring. The aim of this study is to describe the prevalence and patterns of comorbidities among patients with PD in a private periodontal referral practice. **Methods:** This study involved 3171 adults with PD. Data on demographics, lifestyle, number of teeth, pockets of size ≥ 6 mm, bleeding on probing, periodontal inflammatory surface area, and comorbidities were extracted from electronic patient records. Descriptive and statistical analyses, including *t*-tests, chi-square tests, cluster analysis, binomial logistic regression analysis, and hypergraph network analysis, were performed. **Results:** Among this PD population, 47% had a comorbidity, and 20% had multimorbidity (≥2 diseases). Based on the disease patterns, two distinct clusters emerged: Cluster 1 was dominated by respiratory tract conditions (asthma, lung disease, and allergic rhinitis), allergies, and hypothyroidism, while Cluster 2 primarily included cardiometabolic diseases (angina pectoris, hypertension, diabetes mellitus (DM), and hyperthyroidism). The hypergraph network analysis for those with multimorbidity identified two main groups: (i) pulmonary conditions (lung disease, asthma, allergic rhinitis, and allergies) and (ii) cardiometabolic disorders (hypertension, myocardial infarction, cerebrovascular disease, and DM). Hypertension, allergies, and allergic rhinitis showed high centrality, serving as central nodes frequently co-occurring with other diseases. **Conclusions:** Nearly half of the PD patients in a private periodontal referral practice were found to have comorbidities, primarily clustering into cardiometabolic and respiratory tract diseases. These findings, based on real-world data, should encourage dental professionals to integrate systemic conditions into their care strategies. They could also guide policymakers and practitioners in developing evidence-based approaches to mitigate the reciprocal negative effects of PD and comorbidities.

## 1. Introduction

Oral conditions remain a significant global challenge, with studies reporting that around 50–60% of adults have mild-to-moderate periodontitis (PD), and 10–20% have severe PD. In 2021, over 1 billion people worldwide suffered from severe PD, making it a common non-communicable chronic inflammatory condition [1,2,3,4]. In the Netherlands, the prevalence of mild-to-severe PD ranges from 16% to 31% [5,6,7]. Advancing age significantly heightens the risk of PD [3,8]. As Europe’s population ages, the demand for periodontal care will increase, necessitating improved prevention strategies and policy action [8].

The association of PD with diabetes mellitus (DM) and cardiovascular diseases (CVD) is well-documented, and further studies have linked PD to other systemic conditions [9]. The prevalence of comorbidity and multimorbidity is rising in Europe and the Netherlands [10,11]. In a prior study of 37,801 individuals in a dental school setting, patients with PD exhibited a higher prevalence of comorbidity and multimorbidity compared to those without PD, and 46% of the PD patients reported one or more systemic diseases [7].

Dental professionals must consider a patient’s overall health before treatment to minimize risks. Treatment planning should address the reciprocal influence of systemic conditions on PD and its non-surgical and surgical treatments. For example, treatments may need to be postponed for patients with a recent cancer treatment, heart surgery, or immunosuppressive therapy, requiring consultation with medical specialists [12,13,14]. Poor metabolic control in DM patients can impair wound healing and increase periodontal inflammation [9,15,16]. Chronic inflammation is also common in thyroid diseases, which could impact periodontal susceptibility and treatment [9,17]. Given the high likelihood of comorbidities among PD patients, thorough medical histories are essential before treatment, involving coordination with general practitioners and specialists [12,13,14]. Patients with severe PD are typically referred to specialized periodontal clinics. Understanding the prevalence of systemic diseases in these patients could help policymakers and practitioners to develop evidence-based policies for PD management, addressing the comprehensive health needs of these patients [18,19,20].

This study examines the prevalence of comorbidities in patients with PD referred to a specialized non-academic clinic, reflecting real-world practice. It aims to identify the systemic disease patterns in this population and validate the prior findings from a study in a dental school cohort [7].

## 2. Materials and Methods

### 2.1. Study Population, Data Collection, and Data Cleaning

This single-center cross-sectional observational study was registered with the Internal Review Board of the Academic Centre for Dentistry Amsterdam (ACTA) (protocol number 2024-72818) and the reporting followed STROBE guidelines [21]. Informed consent was waived as the data being utilized were pseudonymized and, in accordance with Article 458 of the Dutch Medical Treatment Act (WGBO), obtaining informed consent from individuals was not required. The dental patients were referred by their general dentist to a specialized periodontal clinic (Praktijk voor Parodontologie en Implantologie Nijmegen/Horst, Nijmegen and Horst, The Netherlands) for consultation and treatment planning for PD from 1 January 2017 to 31 December 2022. The referral area of the clinic mainly included the south-eastern part of the country.

At their first appointment at the clinic, the patients were asked to fill out a medical history questionnaire; this questionnaire is provided in the Supplementary Materials available online and is labeled as Table S1. Thereafter, a full periodontal chart was recorded by a periodontist. Each patient was informed about the use of their data for research purposes and was assigned a unique record number which is not retraceable to an individual patient. The data from the medical history questionnaire were verified through an interview and entered into the electronic patient record (EPR) in the clinic’s digital platform (OASE Dental version 3.5.184, VST Software BV, Haarlem, The Netherlands) by the periodontist. The extracted general information included the following: sex (male/female), date of birth, postal code (only the first 4 numbers), and the date of the first appointment at the clinic. The age of the patient was calculated on the date of the first appointment. The Agency for National Statistics in the Netherlands has developed a socioeconomic position (SEP) score for the different postal code areas in the Netherlands based on the financial prosperity, educational level, and recent employment history of private households [22]. Using this national scoring system, postal code data were converted into normalized individual SEP scores ranging from −1.00 to +1.00. A higher SEP score indicates a higher SEP of an individual’s region of residence.

From the periodontal chart, the following data were extracted: number of teeth, total number of pockets of size ≥ 6 mm, proportion of sites with bleeding on probing (BOP), and periodontal inflammatory surface area (PISA). The PISA quantifies the surface area of inflamed (i.e., BOP) pocket epithelium in square millimeters. It is determined by measuring the probing pocket depth at six sites per tooth and assessing BOP. The cumulative pocket surface area with BOP across the entire mouth is then calculated, providing the PISA value as a complete indicator of periodontal inflammatory burden [23]. The PISA calculation was integrated into the EPR in the clinic’s digital platform (OASE Dental). The stages and grades of the PD patients, according to the 2018 classification scheme [24], could not be extracted from the EPR as this system was not implemented in the practice until 2020. The medical history includes all systemic diseases and conditions present at that time and endured before, and information about the lifestyle factor smoking (yes/no) was available. In total, data on 26 diseases or conditions could be extracted (as listed in Table S2). Hereafter, the term systemic diseases will be used to denote all diseases and conditions. The relevant general, dental, and medical information were combined to construct the database. Only adult individuals (≥18 years of age) and patients with a complete medical history questionnaire were included.

### 2.2. Data Analysis

Descriptive analyses were conducted to study and describe the main features of the patient population, as well as to identify patterns and trends within this population. First, general background characteristics and periodontal characteristics were evaluated for the total patient population, for the group of individuals without any comorbidity, and for the group of individuals with at least one comorbidity. Differences between the individuals with and without comorbidity were assessed via independent sample *t*-tests or chi-square tests. The prevalence of zero, one, two, three, four, or five or more systemic diseases, comorbidity (≥1 systemic diseases), multimorbidity (≥2 systemic diseases), and the mean (±SD) number of systemic diseases was calculated. Furthermore, the prevalences of individual systemic diseases were calculated for the total patient population and for the group of individuals with comorbidities.

Next, the possible existence of specific clusters of patients related to the comorbidities present was explored. A two-step cluster analysis was performed using the log-likelihood measure to reveal natural groupings in the dataset, based on the 26 systemic diseases. The two-step clustering approach included an initial pre-clustering step followed by hierarchical agglomerative clustering, as previously described [7,25]. First, the cluster analysis was performed on the total patient population, revealing two clusters: one cluster without comorbidity and another with comorbidity. Next, the cluster analysis was repeated, including only the individuals with comorbidity. General characteristics, periodontal characteristics, and the prevalence of the individual systemic diseases were calculated per cluster, and the differences between the clusters for these items were assessed as described above. Grouping of the systemic diseases within the clusters was performed based on an adaptation of the International Classification of Diseases 11th Revision (ICD-11) [26]. To test the possible influence of the covariables on the variation of prevalence of systemic diseases between the clusters, the adjusted *p*-values obtained from binomial logistic regression after correction for age, sex, SEP, and smoking were calculated for the individual systemic diseases and for the groups of systemic diseases. IBM SPSS version 27.0 (IBM Corp., Armonk, NY, USA) was used for the analyses described above.

Finally, we investigated the coexistence of diseases and relationships within patients with multimorbidity (≥2 systemic diseases) via hypergraph network analysis [27,28]. In a hypergraph, H = (V,E), where V = {v_1_, v_2_, …, v_n_} represents a set of n nodes (diseases in our cohort), and E = {e_1_, e_2_, …, e_m_} represents a set of m hyperedges (disease combinations of individual patients). We constructed the incidence matrix M (n × m), with n rows representing all the diseases in our cohort and m columns representing each individual patient. The adjacency matrix A was derived using A = MM^T^ − D_n_, where M^T^ is the transpose of M and D_n_ is a diagonal matrix with entries equal to the prevalences [29]. For visualization, we focused on hyperedges representing unique combinations of ≥2 diseases, using ≥8 occurrences to balance noise reduction with the exclusion of significant combinations. This captures frequent and clinically relevant disease relationships while minimizing less common, irrelevant combinations. We identified central diseases within the hypergraph by computing disease centrality using the eigenvectors and eigenvalues of A; in particular, the principal eigenvector, corresponding to the largest eigenvalue, was used to determine disease centrality, highlighting the most significant diseases in the graph [30]. These analyses were conducted in Python 3.9.6, using the NumPy 1.24.3, Pandas 2.2.1, Matplotlib 3.7.2, and HyperNetX version 2.2.0 libraries.

## 3. Results

### 3.1. Study Population

**Figure 1:**
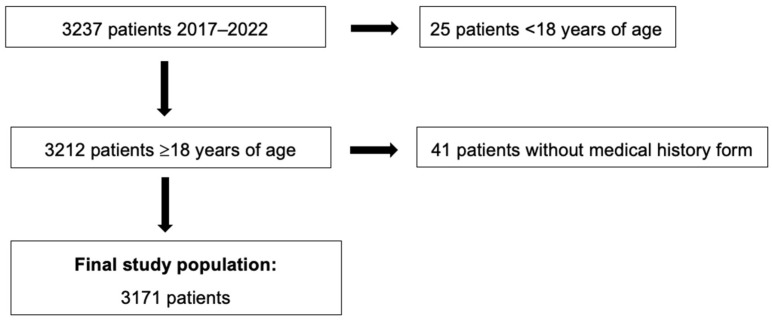
Flowchart of steps to reach the final study population.

Figure 1 presents a flowchart describing how the final study population was reached. Between 1 January 2017 and 31 December 2022, 3237 patients were seen for an intake appointment at the specialized periodontal clinic. Of these, 25 patients were below 18 years of age, and 41 patients declined to fill in the medical history form and were, therefore, excluded from the study population. The final study population included 3171 patients with PD (Figure 1).

### 3.2. General and Periodontal Characteristics

Table 1 details the general and periodontal characteristics of the patients. The mean age was 53 years, with 56% females. The mean SEP score was 0.07 and 25% of the patients were smokers. Periodontal data showed a mean of 26 teeth, 20 pockets of size ≥ 6 mm, a BOP score of 52%, and a PISA of 13 cm^2^. Table 1 also compares patients with (*n* = 1495; 47%) and without comorbidity (*n* = 1676; 53%). Those with comorbidity were more often females (*p* < 0.001) and had significantly fewer teeth (*p* < 0.001). Other characteristics were not significantly different between the two groups.

To explore patterns or clusters of systemic diseases, only patients with comorbidity were included. Two clusters were identified, each presenting groups of patients with certain combinations of systemic diseases. These clusters were significantly different in terms of age (*p* = 0.049), sex (*p* < 0.001), number of teeth (*p* = 0.017), and number of pockets of size ≥ 6 mm (*p* < 0.001), but not in SEP (*p* = 0.36), smoking (*p* = 0.16), BOP (*p* = 0.55), or PISA (*p* = 0.47). Cluster 1 (46%, *n* = 684) had younger patients (mean age: 52), mostly female (68%), fewer pockets of size ≥ 6 mm (mean: 18), and more teeth (mean: 26). Meanwhile, Cluster 2 (54%, *n* = 811) had older patients (mean age: 57), more often male (43%), more pockets of size ≥ 6 mm (mean: 21), and fewer teeth (mean: 25) when compared to cluster 1 (Table 1).

### 3.3. Prevalence of Comorbidity, Multimorbidity, and Number of Systemic Diseases

**Figure 2:**
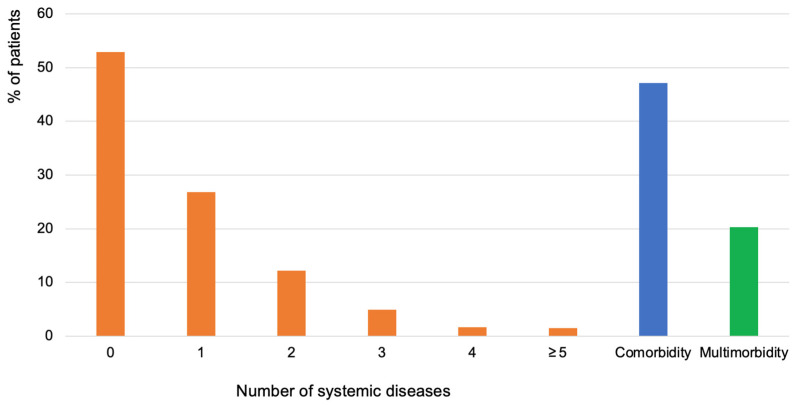
Proportions of patients having systemic diseases (0, 1, 2, 3, 4, ≥5; orange-colored bars), comorbidity (blue-colored bar), and multimorbidity (green-colored bar). No systemic disease (0 on *X*-axis) for *n* = 1676 (53%); 1, 2, 3, 4, and ≥5 systemic diseases for *n* = 851 (27%), *n* = 388 (12%), *n* = 155 (5%), *n* = 55 (1.7%), and *n* = 46 (1.5%), respectively. The prevalence of comorbidity was 47% (*n* = 1495), and that of multimorbidity was 20% (*n* = 644). For the total study population (*n* = 3171), the mean number of systemic diseases was 1.7 ± 1.1 (range = 1–11).

Figure 2 shows the prevalence of the number of systemic diseases, comorbidity, and multimorbidity. The mean number of systemic diseases was 1.7 (SD: 1.1), ranging from 1 to 11; 53% reported having no disease, 27% reported one disease, 12% reported two, 5% reported three, 1.7% reported four, and 1.5% reported at least five diseases. Additionally, 47% had comorbidity and 20% had multimorbidity (Figure 2).

### 3.4. Heterogeneity Among Individuals with PD and Associated Systemic Diseases

Table 2 shows the prevalence of systemic diseases in the total study population, among patients with comorbidity, and within the two clusters. In the total population, the most prevalent diseases were allergy (16%), allergic rhinitis (13%), hypertension (11%), asthma (5%), hypothyroidism (4%), DM (4%), and cardiac arrhythmia (4%). In patients with comorbidity, the prevalences of allergy (35%), allergic rhinitis (28%), hypertension (24%), asthma (10%), hypothyroidism (8%), DM (8%), and cardiac arrhythmia (8%) were higher (Table 2).

Cluster 1 had the highest prevalence of allergy (48%), allergic rhinitis (48%), asthma (15%), lung disease (10%), and hypothyroidism (14%), while Cluster 2 had the highest prevalence of hypertension (44%), DM (14%), cardiac arrhythmia (14%), hyperventilation (13%), myocardial infarction (9%), heart murmur/valve defect (7%), artificial heart valve/pacemaker/hip (7%), cerebrovascular disease (7%), angina pectoris (6%), heart weakness (6%), hyperthyroidism (4%), anemia (4%), chronic gastrointestinal disease (3%), epilepsy (3%), radiation (3%), chronic kidney disease (2%), liver disease (2%), bleeding diathesis (2%), malignant lymph node or blood disease (2%), heart or vascular surgery (1%), and contagious disease (0.5%) (Table 2).

**Figure 3:**
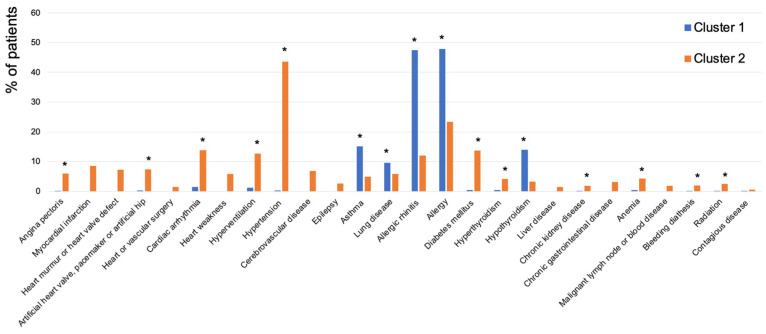
Prevalence of each systemic disease per cluster. * *p*-value comparing the two clusters obtained from binominal logistic regression for all systemic diseases after correction for age, sex, SEP, and smoking, presented as *p* < 0.05 for the significantly highest frequency.

**Figure 4:**
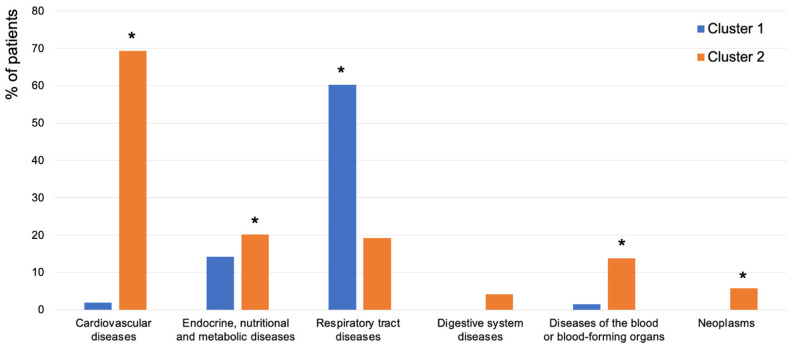
Prevalence of groupings of systemic diseases per cluster. * *p*-value comparing the two clusters for all groups of systemic diseases obtained from binomial logistic regression after correction for age, sex, SEP, and smoking, presented as *p* < 0.05 for the significantly highest frequency. Grouping of the systemic diseases was adapted from the International Classification of Diseases 11th Revision (ICD-11) [28] as follows: cardiovascular diseases—angina pectoris, myocardial infarction, heart murmur or heart valve defect, heart or vascular surgery, cardiac arrhythmia, heart weakness, hypertension, cerebrovascular disease; endocrine, nutritional, and metabolic diseases—diabetes mellitus, hyperthyroidism, hypothyroidism; respiratory tract diseases—asthma, lung disease, allergic rhinitis; digestive system diseases—liver disease, chronic gastrointestinal disease; diseases of the blood or blood-forming organs—anemia, bleeding diathesis; and neoplasms—malignant lymph node or blood disease, radiation.

A binomial logistic regression analysis adjusting for age, sex, SEP, and smoking revealed significant differences between clusters. Cluster 1 had higher prevalences of asthma, lung disease, allergic rhinitis, allergy, and hypothyroidism, while Cluster 2 had higher prevalences of angina pectoris, artificial heart valve/pacemaker/hip, cardiac arrhythmia, hyperventilation, hypertension, DM, hyperthyroidism, chronic kidney disease, anemia, bleeding diathesis, and radiation (Table 2 and Figure 3). Additionally, cluster 2 had significantly higher prevalences of cardiovascular, endocrine, nutritional, metabolic, blood-related diseases, and neoplasms, while Cluster 1 had a higher prevalence of respiratory tract diseases after grouping of the diseases (Figure 4).

### 3.5. Disease Coexistence and Relationships Among Multimorbid Patients

**Figure 5:**
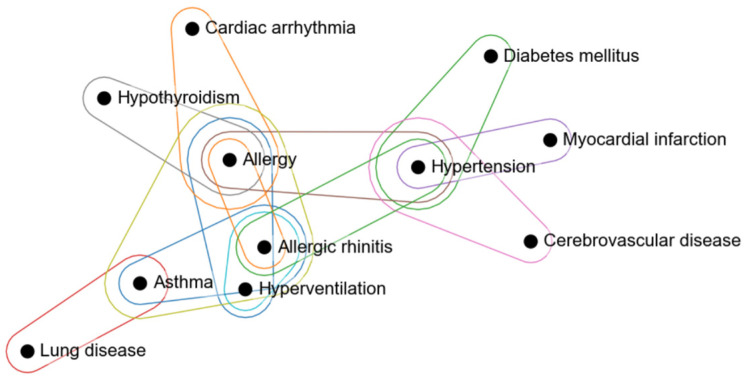
Hypergraph showing disease combinations with eight or more occurrences among patients with multimorbidity (*n* = 644).

Figure 5 visualizes the disease combinations with ≥8 occurrences among patients with multimorbidity (*n* = 644). Each node represents a disease, and hyperedges indicate significant disease relationships. Hypertension, myocardial infarction, cerebrovascular disease, and DM form a cardiometabolic group, while lung disease, asthma, allergic rhinitis, and allergy suggest a respiratory and allergy group (Figure 5). These findings align with our clustering results, highlighting two main PD patient types with comorbidity. Centrality analysis revealed that allergy and allergic rhinitis had the highest centrality, positioning them as central nodes in the hypergraph. Hypertension also emerged as highly central, emphasizing the importance of CVD within the network (Table S3 and Figure S1).

## 4. Discussion

This study demonstrates the prevalence of numerous comorbidities, covering a broad spectrum of systemic diseases, along with their coexistence patterns within a patient population diagnosed with PD. The current study was performed in a non-academic periodontal private practice, using real-world practice data, and confirms our previous findings in a large dental school cohort on comorbidity and multimorbidity trends [7]. On average, individuals in this study had 1.7 systemic diseases, with nearly half reporting one or more comorbidities. Patients with comorbidity were slightly more often female (61% vs. 50%), but there were no differences in terms of the severity of PD between groups. This can be explained, for instance, by noting that certain clusters of comorbidities are associated with increased severity of PD. In particular, Cluster 2 showed a higher prevalence of pockets of size ≥ 6 mm, indicating a greater disease severity. Common systemic diseases included allergy, allergic rhinitis, hypertension, and asthma. Patients with comorbidity fell into two clusters: Cluster 1, presenting mainly respiratory tract diseases (asthma, lung disease, allergic rhinitis) alongside allergy and hypothyroidism; and Cluster 2, presenting mainly cardiometabolic diseases (angina pectoris, hypertension, DM, hyperthyroidism) and conditions such as artificial valve/pacemaker/hip, cardiac arrhythmia, hyperventilation, chronic kidney disease, anemia, bleeding diathesis, and radiation-related issues. Within the multimorbidity group, diseases fell into 2 groups: lung-related ailments (lung disease, asthma, allergic rhinitis, allergy) and cardiometabolic (hypertension, myocardial infarction, cerebrovascular disease, DM). Allergy and allergic rhinitis showed high centrality, which was less known hitherto in relation to other frequent comorbidities with PD. Hypertension also showed high centrality among all the systemic diseases studied in this PD patient population. Hypertension is a predictive factor for future CVD events, in addition to other CVD risk factors [31,32,33].

The study has several strengths. First, it encompassed a substantial sample size (*n* = 3171 patients diagnosed with PD) referred to a specialized periodontal clinic, thus offering a representative sample of a PD population. With this, it should be noted that the patient population does not represent the general Dutch population. Furthermore, comprehensive background data, including date of birth, gender, and postal code, were collected during intake. Additionally, thorough medical information was gathered through the mandatory completion of a medical history questionnaire, which was verified through interviews conducted by the periodontist. Individuals who did not complete the medical history questionnaire (1.3%) were excluded from both the study population and clinical treatment.

The study also had various limitations. While data on age, sex, SEP, and smoking were available and included in the analyses, other potentially important covariates such as body mass index, medication usage, and diet were not included. However, it is plausible that age, SEP, and smoking may serve as surrogate covariates for the unaccounted factors. Another limitation pertains to the self-reported nature of the medical history information provided by patients, which may introduce bias. Nevertheless, the presence of any self-reported disease was corroborated through interviews conducted by the periodontists. Although the questionnaire encompassed a broad range of systemic diseases (26 in total) pertinent to the association with and treatment of PD patients, it did not cover conditions such as rheumatoid arthritis, auto-immune diseases, and neurological disorders, and the question about cancer did not include all cancers and related treatments. Moreover, the question concerning the presence of an artificial heart valve/pacemaker/hip was somewhat ambiguous and not conducive to a straightforward (yes or no) response. Considering the above, the study reveals the shortcomings of the medical history questionnaire, prompting the need for adjustments.

Thus, according to the abovementioned limitations, the current report may seem to have limited generalizability to similar referral periodontal practices as they exist in Europe and beyond. However, the observations in the current study, revealing that 47% of the PD patients in the specialized periodontal clinic exhibited comorbidity, is closely aligned with the rate of 46% observed in PD patients in a dental school [7]. Similarly, the prevalence of multimorbidity among PD patients in both studies was consistent, with the current study indicating 20% and the previous study demonstrating 24% of PD patients experiencing multimorbidity [7]. Furthermore, another study on PD patients identified various systemic multimorbidity clusters [27] and revealed that hypertension was the most common comorbidity (63.9%). PD patients showed a higher likelihood of co-occurring hypertension, obesity, and DM, and this significantly shaped multimorbidity clusters among those with severe PD [27]. In the current study, hypertension had an overall prevalence of 11.2%, but was 43.6% in cluster 2, suggesting that these patients might resemble those in the study of Larvin et al. [27]. Furthermore, the DM prevalence was 14% in cluster 2, compared to 22% in Larvin et al. [27]. The latter study also reported a high smoking prevalence (48%), which can be linked to severe PD and higher rates of hypertension and DM, when compared to the 25% smokers in the current study. Similarly to Larvin et al. [27], we observed the predominance of cardiometabolic diseases, including hypertension, angina pectoris, and DM, in one of the two clusters (cluster 2). Additionally, we showed, through the hypergraph analysis, a group of cardiometabolic diseases connecting hypertension, myocardial infarction, cerebrovascular disease, and DM. Hypertension was highly central, possibly acting as a proxy for and highlighting the importance of CVD in the network, potentially acting as the connection between various serious health issues. Another recent large-scale study has corroborated these findings, identifying hypertension and DM as the most common conditions among PD patients, with hypertension–DM being the most frequent combination, and hypertension, DM, and cardiac rhythm disorders as the most prevalent triad [34]. Although further validation is needed, from the above, we can prudently convey the message that a substantial proportion of PD patients suffer from at least one comorbidity or from multimorbidity. This should be taken into account in daily periodontal practice in order to promote a more holistic approach to patient health.

The existing literature often presents evidence of an association between PD, DM, and CVD [9,35,36]. However, some studies have reported minimal or no effect of PD as an independent risk factor for CVD [6,37] or DM [38]. Our current and previous studies have suggested that certain subsets of PD patients show significant associations with CVD and DM, while others may be linked to respiratory tract diseases. The hypergraph network analysis indicated that allergy and allergic rhinitis have the highest centrality, acting as central nodes that commonly co-occur with other diseases, highlighting their central role in the broader health context of the cohort. Epidemiological studies have revealed a link between oral health and lung diseases, indicating a higher risk of respiratory issues with poor oral health [39,40]. Allergic rhinitis, caused by an allergic reaction to pollen, leads to the release of cytokines and chemokines from mast cells, recruiting inflammatory cells. It affects 5% to 50% of the global population, impacting quality of life and sleep [41]. PD may be linked to allergic diseases through the systemic release of pro-inflammatory cytokines during periods of allergic reactions, inducing hyperactivity of the immune response to oral pathogens. A Korean study found that poor oral health was correlated with allergic rhinitis, asthma, and atopic dermatitis in adolescents [42]. However, another study found an inverse association between PD and allergic rhinitis in adults [43]. A recent study linked asthma to a lower risk of PD; however, this connection seemed to be affected by smoking habits and the use of certain asthma medications, which were associated with a higher risk of PD in asthmatic individuals. Allergies to codeine and latex have also been linked to higher odds of PD [44]. Further research on the relationships between PD and allergic diseases is required.

In clinical practice, patients with CVD or respiratory tract diseases require careful medication management, such as anticoagulants for CVD and inhalation medications (e.g., bronchodilators/corticosteroids and other anti-inflammatories), which can cause dental side effects including cavities, tooth wear, and oral inflammation due to reduced saliva and increased acidity [45]. Proper inhaler use and mouth rinsing can help to mitigate these effects. Poor metabolic control in DM can worsen periodontal inflammation and impair wound healing [16]. Dental professionals should take precautions with patients who have cardiometabolic and respiratory tract diseases and be aware of undiagnosed conditions that could affect oral health.

The association between PD and comorbidities is complex and multifaceted. This study contributes to the existing knowledge by offering a broader perspective, shifting the focus from specific systemic diseases to the prevalence and co-occurrence of various diseases and conditions within PD patient populations. Using real-world data from a periodontal referral practice, this research highlights the patterns of systemic diseases among PD patients. The use of clustering and hypergraph network analyses to group PD patients based on their systemic diseases, identifying two main clusters—cardiometabolic diseases and respiratory tract diseases—represents a significant innovation in this research exploring the link between PD and systemic conditions. Future studies should replicate this approach in diverse patient populations in order to further elucidate the patterns of systemic disease in dental patients and improve the generalizability of the results. Updating and validating medical history questionnaires in dental care should be prioritized to strengthen this research area and contemporary developments should be integrated into dental practices, particularly as the prevalence of non-communicable diseases rises in an increasingly aging population.

Enhancing our understanding of the link between oral and systemic diseases could significantly improve comprehensive care, improve patient outcomes, and potentially reduce long-term healthcare costs. Dental diseases rank as the third most costly health issue in Europe, with an annual cost of EUR 90 billion, following DM and CVD [46]. Dental professionals—especially periodontists and dental hygienists, who regularly see PD patients—are uniquely positioned to identify underlying health issues in those with PD. Based on the findings, a primary focus should be placed on cardiometabolic and respiratory diseases. These professionals can raise health awareness, recommend screenings (e.g., for blood pressure and hyperglycemia), and provide guidance on reducing risk factors such as smoking and a poor diet. Likewise, medical professionals should advocate for an increased awareness of oral health among their patients. A collaborative effort between dental and medical fields can foster a holistic, multidisciplinary approach to healthcare, ultimately improving patient outcomes and reducing costs.

## 5. Conclusions

This study revealed that nearly half of PD patients in a periodontal referral practice had one or more comorbidities, primarily falling within two main clusters: one associated with cardiometabolic diseases and another with respiratory tract diseases. These findings underscore the need for precautionary measures when treating PD patients, emphasizing the importance of understanding their systemic health conditions. The presented results could assist policymakers and practitioners in developing evidence-based strategies to address the comprehensive healthcare needs of PD patients, aiming to mitigate the reciprocal negative effects of PD and systemic diseases with respect to one another.

## Figures and Tables

**Table 1:** Frequencies and prevalence of the general and periodontal characteristics for the total study population, patients without and with comorbidity, and for individual clusters of individuals with periodontitis (PD) and comorbidity.

	Total	No Comorbidity	Comorbidity	Cluster 1	Cluster 2
Number of Cases (%)	3171 (100%)	1676 (52.9%)	1495 (47.1%)	684 (45.8%)	811 (54.2%)
Age	52.9 ± 12.5	51.3 ± 12.3	54.8 ± 12.4	51.7 ± 12.4	57.4 ± 11.8 *
Sex Female	1774 (55.9)	849 (50.7)	925 (61.9) *	465 (68.0) *	460 (56.7)
Male	1397 (44.1)	827 (49.3)	570 (38.1)	219 (32.0)	351 (43.3)
SEP ^§^	0.07 ± 0.19	0.07 ± 0.20	0.06 ± 0.19	0.08 ± 0.18	0.05 ± 0.19
Smoking	791 (24.9)	436 (26.0)	355 (23.7)	151 (22.1)	204 (25.2)
Number of pockets ≥ 6 mm	20.3 ± 21.4	20.9 ± 21.9	19.7 ± 20.8	18.1 ± 19.8	21.1 ± 21.6 *
Bleeding on probing	51.5 ± 25.9	51.5 ± 25.8	51.5 ± 26.1	48.9 ± 25.9	53.6 ± 26.0
Number of teeth	25.7 ± 3.5	26.0 ± 3.4	25.4 ± 3.7 ^†^	25.9 ± 3.5	24.9 ± 3.8 ^†^
PISA	13.4 ± 8.7	13.6 ± 8.8	13.2 ± 8.6	12.7 ± 8.5	13.7 ± 8.6

Abbreviations: SEP, socioeconomic position; PISA, periodontal inflammatory surface area. Values represent numbers (%) or mean in years (±SD). ^§^ Missing data on SEP total: 28 (0.9%), no comorbidity: 13 (0.8%), comorbidity: 15 (1.0%), cluster 1: 10 (1.2%), cluster 2: 5 (0.7%). * Significantly highest mean or frequency after independent sample *t*-test (for the continuous variables: age, SEP, number of pockets of size ≥ 6 mm, BOP, PISA) or chi-square test (for the categorical variables: sex and smoking) between groups with and without comorbidity and between cluster 1 and cluster 2. ^†^ Significantly lower mean after independent sample *t*-test (for the continuous variable: number of teeth) between groups with and without comorbidity and between cluster 1 and cluster 2.

**Table 2:** Frequencies and prevalence of the comorbidities for the two clusters of individuals with PD and comorbidity.

	Total	Comorbidity	Cluster 1	Cluster 2
Number of Cases (%)	3171 (100%)	1495 (47.1%)	684 (45.8%)	811 (54.2%)
Angina pectoris	49 (1.5)	49 (3.3)	1 (0.1)	48 (5.9) *
Myocardial infarction	69 (2.2)	69 (4.6)	0 (0.0)	69 (8.5)
Heart murmur or heart valve defect	59 (1.9)	59 (3.9)	0 (0.0)	59 (7.3)
Artificial heart valve/pacemaker/hip	62 (2.0)	62 (4.1)	2 (0.3)	60 (7.4) *
Heart or vascular surgery ^a^	11 (0.3)	11 (0.7)	0 (0.0)	11 (1.4)
Cardiac arrhythmia	122 (3.8)	122 (8.2)	10 (1.5)	112 (13.8) *
Heart weakness	47 (1.5)	47 (3.1)	0 (0.0)	47 (5.8)
Hyperventilation	111 (3.5)	111 (7.4)	8 (1.2)	103 (12.7) *
Hypertension	356 (11.2)	356 (23.8)	2 (0.3)	354 (43.6) *
Cerebrovascular disease	55 (1.7)	55 (3.7)	0 (0.0)	55 (6.8)
Epilepsy	21 (0.7)	21 (1.4)	0 (0.0)	21 (2.6)
Asthma	143 (4.5)	143 (9.6)	103 (15.1) *	40 (4.9)
Lung disease	112 (3.5)	112 (7.5)	65 (9.5) *	47 (5.8)
Allergic rhinitis	422 (13.3)	422 (28.2)	325 (47.5) *	97 (12.0)
Allergy ^b^	516 (16.3)	516 (34.5)	327 (47.8) *	189 (23.3)
Diabetes mellitus	114 (3.6)	114 (7.6)	3 (0.4)	111 (13.7) *
Hyperthyroidism	37 (1.2)	37 (2.5)	3 (0.4)	34 (4.2) *
Hypothyroidism	122 (3.8)	122 (8.2)	95 (13.9) *	27 (3.3)
Liver disease	12 (0.4)	12 (0.8)	0 (0.0)	12 (1.5)
Chronic kidney disease	16 (0.5)	16 (1.1)	1 (0.1)	15 (1.8) *
Chronic gastrointestinal disease	25 (0.8)	25 (1.7)	0 (0.0)	25 (3.1)
Anemia	38 (1.2)	38 (2.5)	3 (0.4)	35 (4.3) *
Malignant lymph node or blood disease	15 (0.5)	15 (1.0)	0 (0.0)	15 (1.8)
Bleeding diathesis	17 (0.5)	17 (1.1)	1 (0.1)	16 (2.0) *
Radiation ^c^	21 (0.7)	21 (1.4)	1 (0.1)	20 (2.5) *
Contagious disease	5 (0.2)	5 (0.3)	1 (0.1)	4 (0.5)

Values represent numbers (%). ^a^ Within the last 6 months. ^b^ Allergic reaction due to medication or medical material. ^c^ Radiated for tumor in the head or neck. * *p*-value comparing the two clusters for all systemic diseases obtained from binomial logistic regression after correction for age, sex, SEP, and smoking, presented as *p* < 0.05 for the significantly highest frequency.

## Data Availability

The data presented in this study are available on request from the corresponding author due to privacy or ethical restrictions.

## Supplementary Materials

The following supporting information can be downloaded at: https://www.mdpi.com/article/10.3390/jcm13237410/s1, Table S1: Medical History Questionnaire, original version from the Periodontal Referral Practice; Table S2: Extracted systemic diseases and conditions from the Medical History Questionnaire; Table S3: Centrality and prevalence per disease in patients with multimorbidity; Figure S1: Centralities of the systemic diseases within the hypergraph.
