# Supplementary material for: The Prevalence of Comorbidities in Individuals with Periodontitis in a Private Periodontal Referral Practice
Source: J Clin Med. 2024 Dec 5;13(23):7410. doi: 10.3390/jcm13237410 (PMC11642081; doi:10.3390/jcm13237410)
Supplement: Supplementary file 1 [file jcm-13-07410-s001.zip › jcm-3343583-supplementary.pdf]

Table S1. Medical History Questionnaire, original version from the Periodontal Referral Practice.

Questions		Answer
**1**	Do you experience chest pain or a squeezing sensation during strenuous activities? (angina pectoris)? If so,	Yes / No
**2**	Have you ever had a heart attack? If so,	Yes / No
**3.1**	Do you have a heart murmur or heart valve defect? If so,	Yes / No
**3.2**	Do you have an artificial heart valve, pacemaker or artificial hip? If so,	Yes / No
**3.3**	Have you had heart- or vascular surgery in the last 6 months?	Yes / No
**4**	Do you experience cardiac arrhythmia when you are at rest? If so,	Yes / No
**5**	Do you suffer from heart failure? If so,	Yes / No
**6**	Do you have a high blood pressure? If so,	Yes / No
**7**	Have you ever suffered from paralysis (stroke or seizure) or speech impairments? If so,	Yes / No
**8**	Have you ever fainted during dental or medical treatment?	Yes / No
**9**	Are you taking medication for epilepsy? If so,	Yes / No
**10**	Do you suffer from hyperventilation?	Yes / No
**11**	Do you have asthma? If so,	Yes / No
**12**	Do you have bad lungs? If so,	Yes / No
**13.1**	Do you have allergic rhinitis?	Yes / No
**13.2**	Have you ever had an allergic reaction to medication or medical materials? If so, for which?	Yes / No
**14**	Do you have diabetes? If so,	Yes / No
**15**	Have you been diagnosed with enhanced thyroid function? If so,	Yes / No
**16**	Have you been diagnosed with sluggish thyroid function? If so,	Yes / No
**17**	Do you have a liver disease? If so,	Yes / No
**18**	Do you have a chronic kidney disease? If so,	Yes / No
**19**	Do you suffer from chronic gastrointestinal symptoms which caused you to lose more than 11 lbs (5kg)? If so,	Yes / No
**20**	Are you currently suffering from a contagious disease? If so, from which?	Yes / No
**21**	Do you suffer from symptomatic anemia (tired, dizziness)?	Yes / No
**22**	Do you have a malignant disease of the lymph nodes or a blood disorder? If so, which?	Yes / No
**23**	Have you been diagnosed with bleeding tendency? If so,	Yes / No
**24**	Did you undergo radiation therapy for a tumor in the head or neck? If so,	Yes / No
**25**	Are you currently taking any medication, drugs or alcohol?	Yes / No
**26**	Do you smoke? If so, how much?	Yes / No
**27**	Is there a possibility that you are pregnant right now?	Yes / No

Table S2. Extracted systemic diseases and conditions from the Medical History Questionnaire.

	**Systemic diseases**
1.	Angina pectoris
2.	Myocardial infarction
3.	Heart murmur or heart valve defect
4.	Artificial heart valve/pacemaker/hip
5.	Heart or vascular surgery within in the last 6 months
6.	Cardiac arrhythmia
7.	Heart weakness
8.	Hypertension
9.	Cerebrovascular disease
10.	Epilepsy
11.	Hyperventilation
12.	Asthma
13.	Lung disease
14.	Allergic rhinitis
15.	Allergic reaction due to medication or medical material
16.	Diabetes mellitus
17.	Hyperthyroidism
18.	Hypothyroidism
19.	Liver disease
20.	Chronic kidney disease
21.	Chronic gastrointestinal disease
22.	Contagious disease
23.	Anemia
24.	Malignant lymph node or blood disease
25.	Bleeding diathesis
26.	Radiated for tumor in the head or neck

Table S3. Centrality and prevalence per disease in patients with multimorbidity.

**Systemic diseases**	**Centralities**	**Prevalence**
Allergy	0.54	0.50
Allergic rhinitis	0.46	0.36
Hypertension	0.41	0.38
Asthma	0.27	0.17
Lung disease	0.24	0.14
Cardiac arrhythmia	0.24	0.16
Hyperventilation	0.18	0.12
Diabetes mellitus	0.15	0.11
Hypothyroidism	0.14	0.10
Heart weakness	0.13	0.07
Angina pectoris	0.11	0.07
Myocardial infraction	0.10	0.08
Heart murmur or heart valve defect	0.08	0.07
Artificial heart valve/pacemaker/artificial hip	0.08	0.07
Cerebrovascular disease	0.08	0.06
Anemia	0.07	0.04
Chronic gastrointestinal disease	0.05	0.03
Hyperthyroidism	0.04	0.04
Bleeding diathesis	0.03	0.02
Malignancy	0.03	0.02
Chronic kidney disease	0.03	0.02
Heart or vascular surgery	0.03	0.01
Radiation	0.02	0.02
Liver disease	0.01	0.01
Epilepsy	0.01	0.01
Contagious disease	0.01	0.01

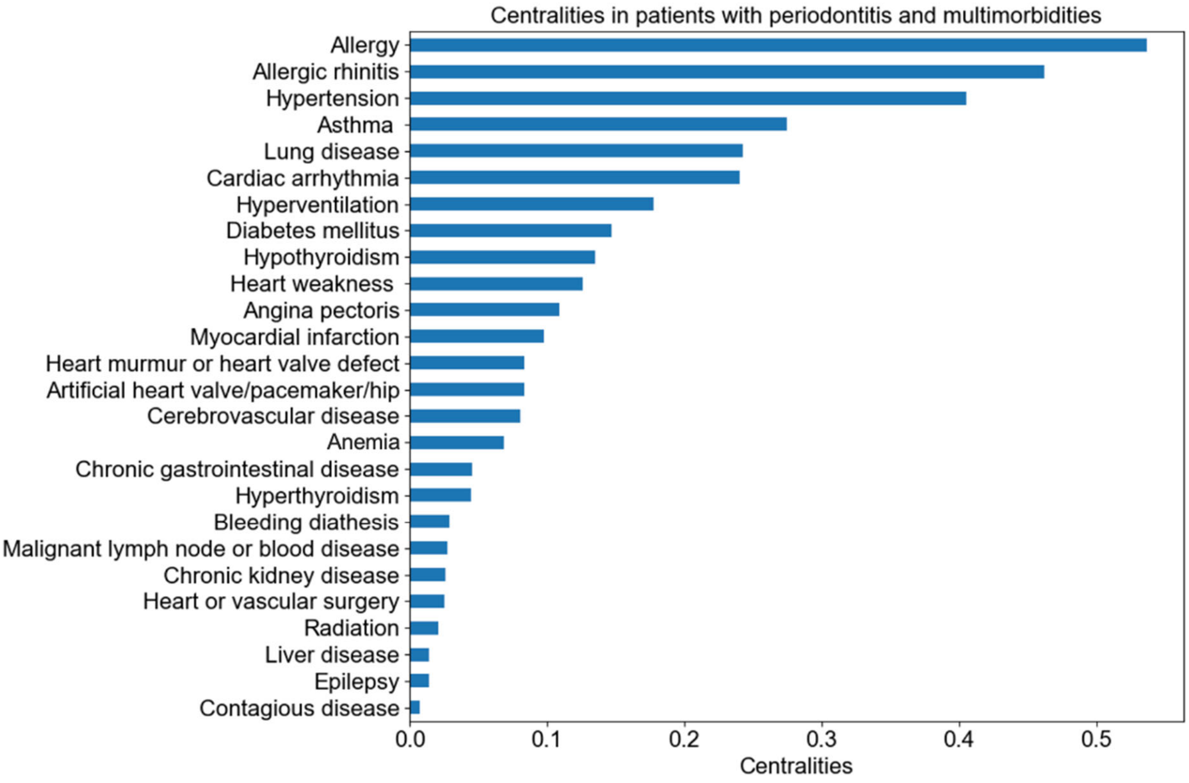

Figure S1. Centralities of the systemic diseases within the hypergraph.
